# Early Alpha-Fetoprotein Response Is Associated With Survival in Patients With HBV-Related Hepatocellular Carcinoma Receiving Lenvatinib

**DOI:** 10.3389/fonc.2022.807189

**Published:** 2022-02-17

**Authors:** Bo Liu, Xiao Shang, Jin-Yu Shi, Guo-Zhen Cui, Xi Li, Nan-Ya Wang

**Affiliations:** The Cancer Center, First Hospital of Jilin University, Changchun, China

**Keywords:** unresectable hepatocellular carcinoma, lenvatinib, AFP response, biomarker, targeted therapy, survival

## Abstract

**Background/Purpose:**

Lenvatinib is a first-line treatment for unresectable hepatocellular carcinoma (uHCC). We assessed the value of early alpha-fetoprotein (AFP) response for predicting clinical outcomes with lenvatinib treatment in patients with HBV-related uHCC and elevated AFP levels.

**Methods:**

This retrospective analysis included patients with HBV-related uHCC and baseline AFP levels ≥20 ng/ml who received lenvatinib for >1 month between November 2018 and May 2021. Early AFP response was defined as a >20% decrease in AFP serum level from baseline after 4 weeks of lenvatinib treatment. Radiological response (Response Evaluation Criteria in Solid Tumors v1.1), progression-free survival, and overall survival were assessed in AFP responders and non-responders.

**Results:**

Of the 46 patients analyzed, 30 (65.2%) were early AFP responders and 16 (34.8%) were non-responders. Compared to the non-responders, early AFP responders had a significantly higher objective response rate (34.5% vs 6.3%, p=0.0349), disease control rate (82.8% vs 50.0%; p=0.0203) and longer median progression-free survival (13.0 vs 7.0 months; HR, 0.464; 95% CI, 0.222-0.967; p=0.028). A subsequent multivariate analysis confirmed that early AFP response (HR, 0.387; 95% CI, 0.183-0.992; p=0.0154), Eastern Cooperative Oncology Group Performance Status of 0 (HR, 0.890; 95% CI, 0.811-0.976; p=0.0132) and Albumin-Bilirubin grade 1 (HR, 0.457; 95% CI, 0.269-0.963; p=0.0327) were independent prognostic factors for longer progression-free survival.

**Conclusion:**

AFP is an important prognostic factor and a predictive biomarker for survival benefit with lenvatinib treatment in patients with HBV-related uHCC.

## Introduction

Worldwide, liver cancer is the sixth most commonly diagnosed cancer, with an estimated 905,677 new cases and 830,180 deaths in 2020 (1). The most common type of liver cancer is hepatocellular carcinoma (HCC), which accounts for 75–85% of cases (2). As HCC is asymptomatic until late in the natural disease course, most patients present with advanced disease, and fewer than 20% of patients are suitable candidates for radical surgery (3). Hence, systemic therapies are the mainstay of treatment for patients with HCC. Recently, systemic treatment options for HCC have expanded, with the emergence of new therapies, particularly targeted agents (4).

Lenvatinib is an oral multikinase inhibitor that targets vascular endothelial growth factor (VEGF) receptors 1-3, fibroblast growth factor (FGF) receptors 1-4, platelet-derived growth factor (PDGF) receptor-α, rearranged during transfection (RET), and KIT, with antiangiogenic and antiproliferative effects (5–7). Findings from clinical and real-world studies have consistently demonstrated the efficacy and good tolerability of lenvatinib in patients with unresectable HCC (uHCC) (8–11). In the REFLECT study, lenvatinib was the first drug to show noninferiority to sorafenib in patients with uHCC with respect to overall survival (OS), as well as statistically significant improvements in progression-free survival (PFS) and objective response rate (ORR) (12). Two thirds of patients in this study were from the Asia-Pacific region and half had hepatitis B virus-related HCC (12). Based on these results, lenvatinib was introduced into clinical practice as a new therapeutic option for the first-line treatment of uHCC (4, 13). However, there remains a need for non-invasive, convenient, and inexpensive biomarkers to assess treatment response and identify which patients are most likely to benefit from lenvatinib therapy.

Serum alpha-fetoprotein (AFP) is a glycoprotein that is overproduced in approximately 70% of patients with HCC (14). AFP levels can be measured using a simple blood test that is routinely available worldwide. Evaluation of changes in AFP levels over time can improve the performance of this biomarker versus a single assessment of the AFP level (15). The association of AFP response with radiological response and prognosis has been assessed in large cohorts of patients with HCC, including those treated with surgical resection, radiofrequency ablation, transarterial chemoembolization, cytotoxic chemotherapy and molecular targeted therapy, and can potentially guide clinical practice in the majority of cases (16–20). For example, previous studies of patients with HCC receiving sorafenib have shown that AFP response is associated with survival, despite differences in AFP criteria for study entry (>20 or >200 ng/ml) and AFP response definitions (21, 22). However, few studies have investigated the value of AFP as a biomarker in patients with advanced HCC treated with lenvatinib (23–25). In particular, a better understanding of the relationship between early AFP response and clinical outcomes in these patients may facilitate decisions on whether to continue lenvatinib treatment.

We performed a retrospective analysis to examine the association between early AFP response and treatment outcomes in patients with advanced HCC receiving lenvatinib in real-world clinical practice.

## Patients and Methods

### Study Population

We retrospectively reviewed medical records of consecutive patients with HBV-related uHCC who received lenvatinib between November 2018 and May 2021 at the Cancer Center of the First Hospital of Jilin University. Inclusion criteria were: uHCC diagnosed using contrast enhanced computed tomography (CT), magnetic resonance imaging (MRI), or tumor biopsy; baseline AFP level ≥20 ng/ml; and lenvatinib treatment duration of ≥1 month. The study was conducted in accordance with the recently revised Declaration of Helsinki and approved by the ethics committee in our institution. Written informed consent was waived for this study because of the retrospective nature of the study. AFP assessment and definition of early AFP response

Serum AFP levels were measured at baseline (before the administration of lenvatinib) and at 1 month after starting lenvatinib therapy. There are no standardized cutoff values for AFP response, although thresholds of 20% and 50% change from baseline are frequently used (23, 24). In this study, early AFP response was defined as a >20% decrease in serum AFP levels after 1 month of lenvatinib treatment.

### Treatment and Outcome Assessments

All patients received oral lenvatinib at a dose of 8 mg/day (bodyweight <60 kg) or 12 mg/day (bodyweight ≥60 kg). According to the instructions for lenvatinib administration, the dose was reduced or treatment was interrupted if grade ≥3 adverse events (AEs) or any unacceptable grade 2 AEs occurred. If a drug-related grade ≥3 AE or unacceptable grade 2 AE occurred, dose reduction or temporary interruption was maintained until the AE improved to grade 1 or 2. AEs were assessed using the National Cancer Institute Common Terminology Criteria for Adverse Events, version 4.0.

Radiological evaluations using enhanced CT or MRI were performed after 1 month of lenvatinib treatment and every 2-3 months thereafter, or whenever there was a sign or symptom suggesting tumor progression. Radiological response was determined according to the Response Evaluation Criteria in Solid Tumors (RECIST) v1.1. ORR was defined as the percentage of patients who achieved complete response (CR) or partial response (PR). The disease control rate (DCR) was defined as the percentage of patients who achieved a CR, PR or stable disease (SD). PFS was defined as the time from the start of lenvatinib treatment until tumor progression or death. OS was defined as the time from the start of lenvatinib treatment until death from any cause.

### Statistical Analysis

Continuous variables were summarized using median values with interquartile (IQR) ranges, and intergroup values were compared using Mann–Whitney U tests. Categorical variables were summarized as number and percentage and were compared using a Fisher’s exact test. PFS and OS were calculated using the Kaplan–Meier method and intergroup differences compared using a log–rank test. Radiological responses, PFS, and OS were assessed in all patients and stratified by early AFP responders and AFP non-responders. Potential prognostic factors for PFS and OS were assessed using univariate and multivariate Cox proportional hazards models. All factors exhibiting a significant association with PFS or OS in the univariate analyses were included in the multivariate models. For subgroup analyses of PFS and OS, a univariate Cox proportional hazard model was used to estimate the hazard ratios (HRs) and corresponding 95% confidence intervals (CIs) for AFP responders versus AFP non-responders in specific patient subgroups. For all analyses, p<0.05 was considered statistically significant and all reported p values are two-sided. All statistical analyses were performed using R software version 3.6.3 (http://www.R-project.org/).

## Results

### Patients

Medical records from a total of 79 patients were screened, of whom 46 were included in the analysis. Reasons for exclusion were diagnosis of non-HBV related HCC (n=4), baseline AFP <20 ng/ml (n=29). At baseline, more than half of patients (65.2%) were classified as Child-Pugh class A. Extrahepatic metastases were present in 52.2% of patients and portal vein thrombosis was noted in 34.8%. The median tumor size was 5.6 cm, with 17.4% and 80.4% of patients classified as Barcelona Clinic Liver Cancer (BCLC) stages B and C, respectively. In total, 82.6% of patients had received lenvatinib as first-line therapy, and the patients in the second-line setting are all progressed after sorafenib. The median follow-up period was 18.0 (IQR, 3-29) months. Overall, 10 (21.7%) patients had a lenvatinib dose reduction and three (6.5%) had a treatment interruption due to AEs.

Of the 46 patients analyzed, 30 (65.2%) were early AFP responders and 16 (34.8%) were AFP non-responders. Median baseline AFP levels were 660.25 ng/ml and 1199.0 ng/ml in early AFP responders and non-responders, respectively. Baseline characteristics were similar between early AFP responders and non-responders, except for age (p=0.0336), portal vein thrombosis (p=0.0205) and ascites(p=0.04) (**Table 1**).

**Table 1:** Baseline characteristics by AFP response.

	AFP response (N = 30)	AFP none response (N = 16)	p value
Gender, male/female			
Male	26 (86.7%)	16 (100%)	0.1264
Female	4 (13.3%)	0	
Age, ≥60/<60 years			
≥60	13 (43.3%)	2 (12.5%)	0.0336
<60years	17 (56.7%)	14 (87.5%)	
Baseline AFP,ng/mL			
n (nmiss)	30 (0)	16 (0)	
Median	660.25	1199.0	0.8717
AST,U/L			
n (nmiss)	30 (0)	16 (0)	
Median	48.50	33.15	0.1195
ALT,U/L			
n (nmiss)	30 (0)	16 (0)	
Median	40.40	26.75	0.0922
Total bilirubin, μmol/L			
n (nmiss)	30 (0)	16 (0)	
Median	22.95	19.45	0.4262
Albumin, g/L			
n (nmiss)	30 (0)	16 (0)	
Median	37.10	39.90	0.4745
Platelet count,×10^9^/L			
n (nmiss)	29 (1)	14 (2)	
Median	114.00	128.50	0.3643
Hemoglobin, g/L			
n (nmiss)	29 (1)	14 (2)	
Median	146.00	139.00	0.0656
Prothrombin time, s			
n (nmiss)	27 (3)	14 (2)	
Median	12.40	11.90	0.1645
International normalized ratio			
n (nmiss)	27 (3)	14 (2)	
Median	1.10	1.03	0.2207
BCLC stage B/C			
A	0	1 (6.3%)	0.1512
B	7 (23.3%)	1 (6.3%)	
C	23 (76.7%)	14 (87.5%)	
Maximum tumor diameter, cm			
n (nmiss)	30 (0)	16 (0)	
Median	6.00	3.60	0.1451
Number of tumors, solitary/multiple			
Null	2 (6.7%)	3 (18.8%)	0.451
solitary	8 (26.7%)	4 (25.0%)	
multiple	20 (66.7%)	9 (56.3%)	
Extrahepatic metastasis, yes/no			
Yes	14 (46.7%)	2 (12.5%)	0.0205
No	16 (53.3%)	14 (87.5%)	
Portal vein thrombosis, yes/no			
Yes	14 (46.7%)	2 (12.5%)	0.0205
No	16 (53.3%)	14 (87.5%)	
Cirrhosis, yes/no			
Yes	24 (80.0%)	9 (56.3%)	0.0884
No	6 (20.0%)	7 (43.8%)	
Ascites, yes/no			
Yes	17 (56.7%)	4 (25.0%)	0.04
No	13 (43.3%)	12 (75.0%)	
Lenvatinib as first line treatment, yes/no			
Yes	27 (90.0%)	11 (68.9%)	0.1459
No	3 (10.0%)	4 (25.0%)	
Unknown	0 (0)	1 (6.3%)	
ECOG PS 0/1,n (%)			
0	17 (56.7)	10 (62.5)	0.2903
1	13 (43.3)	6 (37.5)	
ALBI grade 1/2			
<=-2.6	9 (30.0)	8 (50.0%)	0.1233
-2.6~-1.39	21 (70.0)	7 (43.8%)	
>-1.39		1 (6.3%)	
Child-Pugh class A/B			
A	21 (70.0%)	9 (56.2%)	0.2709
B	9 (30.0%)	7 (43.8%)	

ALBI, albumin-bilirubin; AFP, alpha-fetoprotein; BCLC, Barcelona Clinic Liver Cancer; ECOG PS, Eastern Cooperative Oncology Group performance status; SD, standard deviation.

### Relationship Between Early AFP Response and Imaging Response

No patients achieved a CR. Among early AFP responders, PR, SD, and PD were observed in 10 (34.5%), 14 (48.3%), and 5 (17.2%) patients, respectively, compared with 1 (6.3%), 7 (43.8%), and 8 (50.0%) patients, respectively, in the non-responder group. Early AFP responders had a significantly higher ORR (34.5% vs 6.3%; p=0.0349) and DCR (82.8% vs 50.0%; p=0.0203) versus non-responders (**Table 2**).

**Table 2:** Relationship between early AFP response and imaging response.

n (%)	Early AFP response (n = 29*)	AFP non-response (n = 16)	p value
Imaging response			
CR	0	0	
PR	10 (34.5)	1 (6.3)	
SD	14 (48.3)	7 (43.8)	
PD	5 (17.2)	8 (50.0)	
ORR			
Yes	10 (34.5)	1 (6.3)	0.0349
DCR			
Yes	24 (82.8)	8 (50.0)	0.0203

AFP, alpha-fetoprotein; CR, complete response; DCR, disease control rate; ORR, objective response rate; PD, progressive disease; PR, partial response; SD, stable disease.

*One patient lacked further imaging examination at data cut-off.

### Relationship Between Early AFP Response and Survival Outcome

**Figure 1:**
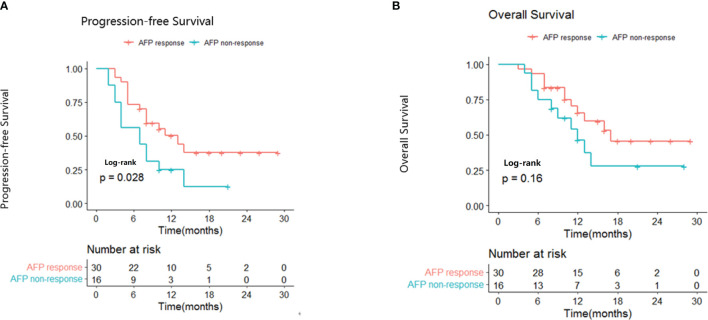
Kaplan-Meier curves for progression-free survival **(A)** and overall survival **(B)** in AFP responders and non-responders.

Early AFP responders had a significantly longer median PFS compared with non-responders (13.0 vs 7.0 months; p=0.028; **Figure 1A**). The results of univariate and multivariate analyses for PFS are presented in **Table 3**. In the univariate analysis, patients were more likely to have longer PFS if they had an early AFP response (HR, 0.464; 95% CI, 0.222-0.967; p=0.0404), ECOG PS of 0 (HR, 0.431; 95% CI 0.265-0.897; p=0.0398) and ALBI grade 1 (HR, 0.538; 95% CI, 0.290-0.973; p=0.0462).A subsequent multivariate analysis confirmed that early AFP response (HR, 0.387; 95% CI, 0.183-0.992; p=0.0154), Eastern Cooperative Oncology Group Performance Status of 0 (HR, 0.890; 95% CI, 0.811-0.976; p=0.0132) and Albumin-Bilirubin grade 1 (HR, 0.457; 95% CI, 0.269-0.963; p=0.0327) were independent prognostic factors for longer progression-free survival.

**Table 3:** Univariate and multivariate analysis for PFS.

	HR	95% CI	p value
**Univariate analysis**			
Age, ≥60/<60 years	0.781	0.355-1.718	0.5386
Early AFP response, yes/no	0.464	0.222-0.967	0.0404
Baseline AFP, ≥400/<400 ng/ml	1.414	0.681-2.935	0.3530
Ascites, yes/no	1.377	0.664-2.853	0.3897
Cirrhosis, yes/no	1.127	0.497-2.555	0.7746
Maximum tumor diameter, ≥10/<10 cm	0.420	0.143-1.235	0.1150
Number of tumors, solitary/multiple	0.793	0.332-1.893	0.6014
Extrahepatic metastasis, yes/no	1.585	0.633-3.972	0.3255
Portal vein thrombosis, yes/no	0.825	0.374-1.819	0.6338
BCLC stage, B/C	0.711	0.270-1.872	0.4903
ECOG PS, 0/1	0.431	0.265-0.897	0.0398
ALBI grade, 1/2	0.538	0.290-0.973	0.0462
Child-Pugh class, A/B	0.658	0.311-1.393	0.2742
**Multivariate analysis**			
Age, ≥60/<60 years	0.535	0.191-1.499	0.2341
Early AFP response, yes/no	0.387	0.183-0.992	0.0154
ECOG PS, 0/1	0.489	0.411-0.976	0.0132
Extrahepatic metastasis, yes/no	1.659	0.525-3.517	0.5271
Portal vein thrombosis, yes/no	1.059	0.437-2.324	0.6574
ALBI grade, 1/2	0.457	0.269-0.963	0.0327

ALBI, albumin-bilirubin; AFP, alpha-fetoprotein; BCLC, Barcelona Clinic Liver Cancer; CI, confidence interval; ECOG PS, Eastern Cooperative Oncology Group performance status; HR, hazard ratio.

Median OS was 17.0 months for early AFP responders compared with 12.0 months for non-responders (p=0.16; **Figure 1B**). The results of univariate and multivariate analyses for OS are presented in **Table 4**. In the univariate analysis, patients were more likely to have longer OS if they had ECOG PS of 0 (HR, 0.531; 95% CI, 0.324-0.991; p=0.0498) and ALBI grade 1 (HR, 0.434; 95% CI 0.258-0.855; p=0.0320). Multivariate analysis showed that ECOG PS of 0 (HR, 0.479; 95% CI, 0.314-0.876; p=0.0332), and ALBI grade 1 (HR, 0.551; 95% CI, 0.160-0.897; p=0.0346) were independent prognostic factors for longer OS.

**Table 4:** Univariate analysis for OS.

	HR	95% CI	p value
**Univariate analysis**			
Age, ≥60/<60 years	1.147	0.488-2.695	0.7537
Early AFP response, yes/no	0.556	0.240-1.288	0.1710
Baseline AFP, ≥400/<400 ng/ml	1.418	0.609-3.302	0.4183
Ascites, yes/no	1.743	0.744-4.086	0.2011
Cirrhosis, yes/no	1.248	0.486-3.203	0.6449
Maximum tumor diameter, ≥10/<10 cm	0.811	0.185-3.550	0.7808
Number of tumors, solitary/multiple	0.559	0.202-1.552	0.2646
Extrahepatic metastasis, yes/no	1.805	0.598-5.445	0.2946
Portal vein thrombosis, yes/no	0.941	0.380-2.326	0.8946
BCLC stage, B/C	0.517	0.152-1.763	0.2921
ECOG PS, 0/1	0.531	0.324-0.991	0.0498
ALBI grade, 1/2	0.434	0.258-0.855	0.0320
Child-Pugh class, A/B	0.718	0.301-1.711	0.4544
**Multivariate analysis**			
Age, ≥60/<60 years	0.900	0.295-2.746	0.8526
Early AFP response, yes/no	0.734	0.229-2.351	0.6023
ECOG, 0/1	0.479	0.314-0.876	0.0332
Extrahepatic metastasis, yes/no	1.934	0.952-3.751	0.2930
Portal vein thrombosis, yes/no	0.948	0.315-2.858	0.9246
ALBI grade, 1/2	0.551	0.160-0.897	0.0346

ALBI, albumin-bilirubin; AFP, alpha-fetoprotein; BCLC, Barcelona Clinic Liver Cancer; CI, confidence interval; ECOG PS, Eastern Cooperative Oncology Group performance status; HR, hazard ratio.

### Relationship Between Liver Function and Survival Outcome

**Figure 2:**
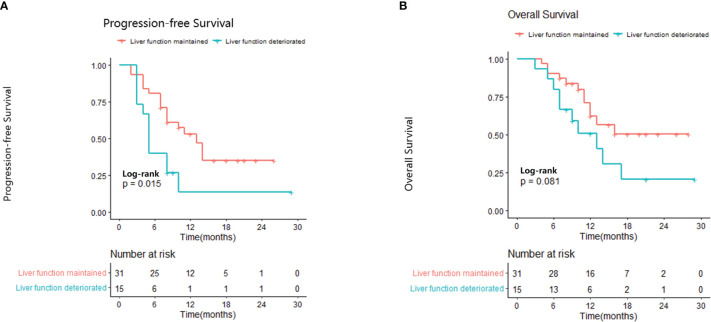
Relationship between liver function and progression-free survival **(A)** and overall survival **(B)**.

We evaluated the association between AFP response and change in liver function. Deterioration of liver function was defined as a change from Child-Pugh class A to Child-Pugh class B after initiating lenvatinib treatment or, in the case of patients with Child-Pugh class B7, the patient’s Child-Pugh score increased to ≥8 points. Most patients (31/46, 67.4%) showed maintained or improved liver function during lenvatinib therapy. There was no significant association between AFP response and change in liver function (p=0.42). Median PFS was significantly longer in patients with maintained or improved liver function compared with those whose liver function deteriorated during treatment (13.0 months vs 5.0 months; p=0.015; **Figure 2A**). Median OS was not reached in the maintained or improved liver function group and was 13 months in the deteriorated liver function group (p=0.081; **Figure 2B**).

## Discussion

Findings from this study in patients with uHCC receiving lenvatinib showed that early AFP responders achieved a significantly higher ORR and DCR compared with AFP non-responders. These results are consistent with previous studies of the relationship between early AFP decline and radiological response in patients with uHCC treated with lenvatinib (23–25). In one study, patients with a sustained reduction of AFP from 2 to 4 weeks after lenvatinib initiation had a higher ORR compared with patients with a non-sustained reduction (67% vs 0%; p=0.02) (24). Another study reported that, among patients with baseline AFP ≥10 ng/ml, AFP responders (defined as those with an AFP reduction of ≥40%) versus non-responders had a significantly higher ORR (68.4% vs 7.1%; p<0.001) and DCR (84.2% vs 36.0%; p=0.009), and AFP response was the only significant predictor of objective response (odds ratio, 51.389; 95% CI, 4.888-540.281; p=0.001) (23). In addition, a multivariate analysis showed that a decrease in AFP level was an independent factor associated with response to lenvatinib (adjusted odds ratio, 10.3; 95% CI, 1.81-58.7; p<0.01) (25). Our results, together with this previous evidence, suggest that early AFP non-response can help to identify patients who are less likely to respond to lenvatinib and may therefore require more frequent imaging assessments.

Moreover, surgical resection remains the most important radical treatment for patients with liver cancer. For patients with advanced liver cancer who do not have an opportunity for surgery, transformational resection (resection after the cancer is reduced or downgraded) has a key role (26). From the perspective of transformational resection, the ORR is one of the most important considerations in the systemic treatment plan (26). However, efficacy predictors for the systemic treatment of liver cancer are lacking. Our research and previous studies have found that patients achieving an early AFP response have a significantly better tumor response compared with non-responders. Therefore, early AFP response may have value in guiding systemic treatment prior to transformational resection.

AFP response has previously been associated with survival in studies of patients with uHCC treated with drugs other than lenvatinib (21, 27). In patients treated with immune checkpoint inhibitors (nivolumab or pembrolizumab), early AFP response (>10% reduction in AFP within 4 weeks of treatment) predicted better objective response and survival (27). Early AFP response was also a significant independent predictor for better PFS and OS following antiangiogenic systemic therapy (21). Furthermore, similar results have been reported in patients receiving locoregional treatments, despite different AFP entry criteria and definitions of AFP response (28, 29). These previous reports support our findings that early AFP response may be a useful predictive marker for survival in patients with uHCC receiving lenvatinib, thereby helping to identify patients with a better prognosis and those who are candidates for other therapy options. The ability of AFP response to predict outcomes in uHCC may be explained by the role of AFP in promoting the growth, proliferation, and metastasis of HCC, and eliciting the escape of HCC from immune surveillance (14, 30, 31). Until now, no studies have investigated the value of AFP response in predicting the survival of patients with uHCC receiving lenvatinib. Our study demonstrated that early AFP responders achieved significantly longer PFS compared with non-responders. In addition, early AFP response was an independent prognostic factor for longer PFS in multivariate analysis. However, our results were inconclusive on whether early AFP response was predictive of OS, as the p-value was not significant in the univariate analysis(HR, 0.556; 95% CI, 0.240-1.288; p=0.1710). This may be related to the limited sample size and short follow-up time.

Previous studies have shown that in some patients with grade 2 albumin-bilirubin at baseline, a decrease in tumor burden in response to treatment leads to an improvement in liver function (32). However, in our study, there was no significant association between AFP response and liver function, which may be related to patient selection. Patients with advanced HCC treated with lenvatinib have previously been shown to have maintained or improved liver functional reserves after 4 and 12 weeks (33). Consistent with this observation, approximately two thirds of patients in the present study had maintained or improved liver function during lenvatinib therapy. As expected, patients with stable liver function had better survival compared with those with worsening liver function.

This study has several limitations. Firstly, it was a retrospective study with a modest sample size and, consequently, the findings require confirmation in further studies with larger numbers of patients. Secondly, as only patients with elevated AFP levels (serum AFP ≥20 ng/ml) at the initiation of lenvatinib therapy were included, the results may not be applicable to patients whose baseline AFP levels are within the normal range. Thirdly, the etiology of HCC in this study was hepatitis B virus (HBV), with a higher proportion of patients with HBV infection than in previous studies conducted in Western countries. As HBV-related hepatitis or cirrhosis may contribute to elevation of AFP, further validation is needed.

In conclusion, early AFP response may be a useful predictor of better tumor response and longer PFS and OS in patients with uHCC receiving lenvatinib. Therefore, early AFP response should be taken into consideration when assessing treatment response to lenvatinib in patients with uHCC, particularly in those with elevated AFP levels prior to treatment initiation.

## Data Availability Statement

The original contributions presented in the study are included in the article/supplementary material. Further inquiries can be directed to the corresponding author.

## Ethics Statement

The studies involving human participants were reviewed and approved by Ethics Committee of the First Hospital of Jilin University Affiliation: First Hospital of Jilin University. Written informed consent for participation was not required for this study in accordance with the national legislation and the institutional requirements.

## Author Contributions

BL and XS drafted the manuscript and contributed for the data analysis. J-YS, G-ZC, and XL critically revised the article. N-YW contributed for study concept and design. All authors read and approved the final manuscript, and all authors have taken due care to ensure the integrity of the work.

## Funding

Jilin Provincial Department of Education grant number 3D5196776428 and Beijing Medical and Health Public Welfare Foundation grant number 3R2205401428.

## Conflict of Interest

The authors declare that the research was conducted in the absence of any commercial or financial relationships that could be construed as a potential conflict of interest.

## Publisher’s Note

All claims expressed in this article are solely those of the authors and do not necessarily represent those of their affiliated organizations, or those of the publisher, the editors and the reviewers. Any product that may be evaluated in this article, or claim that may be made by its manufacturer, is not guaranteed or endorsed by the publisher.
